# A Digital System (YouXin) to Facilitate Self-Management by People With Psychosis in China: Protocol for a Nonrandomized Validity and Feasibility Study With a Mixed Methods Design

**DOI:** 10.2196/45170

**Published:** 2023-09-12

**Authors:** Xiaolong Zhang, Shôn Lewis, Lesley-Anne Carter, Sandra Bucci

**Affiliations:** 1 Division of Psychology and Mental Health School of Health Sciences, Faculty of Biology, Medicine and Health, Manchester Academic Health Science Centre The University of Manchester Manchester United Kingdom; 2 The National Clinical Research Center for Mental Disorders & Beijing Key Laboratory of Mental Disorders Beijing Anding Hospital Capital Medical University Beijing China; 3 Greater Manchester Mental Health NHS Foundation Trust Manchester United Kingdom; 4 Division of Population Health, Health Services Research & Primary Care School of Health Sciences, Faculty of Biology, Medicine and Health, Manchester Academic Health Science Centre The University of Manchester Manchester United Kingdom

**Keywords:** psychosis, self-management, digital, smartphone app, eHealth, mHealth, China

## Abstract

**Background:**

Psychosis is one of the most disabling mental health conditions and causes significant personal, social, and economic burden. Accurate and timely symptom monitoring is critical to offering prompt and time-sensitive clinical services. Digital health is a promising solution for the barriers encountered by conventional symptom monitoring approaches, including accessibility, the ecological validity of assessments, and recall bias. However, to date, there has been no digital health technology developed to support self-management for people with psychosis in China.

**Objective:**

We report the study protocol to evaluate the validity, feasibility, acceptability, usability, and safety of a symptom self-monitoring smartphone app (YouXin; Chinese name 佑心) for people with psychosis in China.

**Methods:**

This is a nonrandomized validity and feasibility study with a mixed methods design. The study was approved by the University of Manchester and Beijing Anding Hospital Research Ethics Committee. YouXin is a smartphone app designed to facilitate symptom self-monitoring for people with psychosis. YouXin has 2 core functions: active monitoring of symptoms (ie, smartphone survey) and passive monitoring of behavioral activity (ie, passive data collection via embedded smartphone sensors). The development process of YouXin utilized a systematic coproduction approach. A series of coproduction consultation meetings was conducted by the principal researcher with service users and clinicians to maximize the usability and acceptability of the app for end users. Participants with psychosis aged 16 years to 65 years were recruited from Beijing Anding Hospital, Beijing, China. All participants were invited to use the YouXin app to self-monitor symptoms for 4 weeks. At the end of the 4-week follow-up, we invited participants to take part in a qualitative interview to explore the acceptability of the app and trial procedures postintervention.

**Results:**

Recruitment to the study was initiated in August 2022. Of the 47 participants who were approached for the study from August 2022 to October 2022, 41 participants agreed to take part in the study. We excluded 1 of the 41 participants for not meeting the inclusion criteria, leaving a total of 40 participants who began the study. As of December 2022, 40 participants had completed the study, and the recruitment was complete.

**Conclusions:**

This study is the first to develop and test a symptom self-monitoring app specifically designed for people with psychosis in China. If the study shows the feasibility of YouXin, a potential future direction is to integrate the app into clinical workflows to facilitate digital mental health care for people with psychosis in China. This study will inform improvements to the app, trial procedures, and implementation strategies with this population. Moreover, the findings of this trial could lead to optimization of digital health technologies designed for people with psychosis in China.

**International Registered Report Identifier (IRRID):**

DERR1-10.2196/45170

## Introduction

### Background

Psychosis is one of the most disabling mental health conditions and causes significant personal, social, and economic burden [1], which increases the risk of physical health problems and reduces life expectancy by around 20 years [2]. Within 5 years after the first episode, 80% of people with psychosis will have at least one relapse [3]. The prevalence of schizophrenia in China is the highest in the world [4]. A recent Chinese national survey showed that the lifetime prevalence rate of schizophrenia and other psychotic disorders was 0.7% [5], and schizophrenia accounted for 13.7% of all mental and substance disorder disability-adjusted life years in 2019 [6].

Accurate and timely symptom monitoring is arguably one of the essential components of quality clinical services. Conventional symptom monitoring approaches in mental health services face several practical and methodological challenges. First, conventional methods rely on clinical interviews or comprehensive assessment batteries, which can be carried out only by trained professionals and require service users to attend an in-person meeting with their health provider. However, a lack of resources means that access to in-person services is limited; the wait times between referral and assessment can range from 4 weeks to 6 months or longer [7]. Meanwhile, organizational barriers (eg, financial availability, transportation) and attitudinal barriers (eg, stigma, reluctance to seek help) prevent people with mental health problems from attending appointments regularly [8]. Moreover, accessing in-person services has been further hindered by the COVID-19 pandemic, since clinic visits have been restricted to mitigate virus transmission [9,10].

Second, the conventional approach to assessing symptoms lacks ecological validity; current methods for assessing service users’ difficulties, broadly speaking, capture cross-sectional information rather than continuous information over a certain period of time [11,12]. Clinical visits tend to be scheduled mostly, at best, monthly, which means health care providers only get a snapshot of service users' conditions every 2 weeks to 6 weeks. However, clinically meaningful symptom fluctuation, such as mood or hallucinations, may occur on a daily basis [13], which conventional methods struggle to capture.

Third, recall bias has long been identified as a limitation of conventional clinical assessment methods [11,14]. Service users, especially people with severe mental health problems for which cognitive impairments are common [15], may have trouble accurately recalling their thoughts, feelings, and behaviors over a preceding period of time, making recall experiences at a granular level challenging [16]. Therefore, to optimize the quality of assessment and capture symptom fluctuation in a real-time, real-world granular manner, more ecologically valid assessment methods are needed.

The aforementioned barriers affect countries worldwide [14], even high-income countries with sophisticated mental health service systems (eg, the United Kingdom and the United States) [17,18], and China has been no exception to these challenges.

### Mental Health Services in China

China has invested substantial resources to reform its mental health system to improve the accessibility and quality of mental health services in the past 15 years; however, the effort has only been partially successful, and the mental health treatment gap remains significant [19,20]. The estimated shortage of mental health professionals in China was 40,000 relative to the population need [21]. According to the WHO Global Health Observatory, as of 2015, there were only 2.20 psychiatrists, 5.42 mental health nurses, and 0.07 mental health hospitals per 100,000 population in China [22]. In contrast, in the United States, there are 10.54 psychiatrists, 4.28 mental health nurses, and 0.19 mental hospitals per 100,000 population [22]. Moreover, since the Chinese health care system is designed as hospital-centered care [23], most well-trained mental health care professionals (ie, psychiatrists, mental health nurses, and psychologists) are concentrated in psychiatric hospitals located in urban areas, which makes it difficult for people in rural or remote communities to access care [24,25].

In China, mental health services are primarily delivered through psychiatric hospitals [26-28]. Compared with other settings (ie, general hospitals and community-based settings), tertiary psychiatric hospitals are best placed to provide quality mental health services, which results in service users overwhelmingly concentrated in tertiary psychiatric hospitals causing long wait times and reduced service quality [26].

### The Role of Digital Health

Digital health is a promising solution to address both the barriers encountered by the conventional symptom monitoring approach [14] and China’s health care system that has a shortage of mental health care resources [29,30]. The ubiquitous accessibility of digital technologies (eg, smartphones) provides unprecedented potential to scale-up service provision. Smartphone ownership rates in China have reached 96% [31]. Previous studies showed that smartphone ownership by people with psychosis was comparable with that of the general population [32]. More specifically, the internet access rate reached 90.9% of people with a diagnosis of schizophrenia in Hong Kong [33], and people with mental health problems in China in general were willing to accept mobile health interventions and considered them helpful [34]. Integrating digital health technologies (DHTs) into clinical workflows provides the potential to augment evidence-based care, support shared decision-making, and increase continuity of care [35]. These potential benefits are true for all countries worldwide and arguably even more so for countries with less sophisticated mental health care systems such as China. We conducted a systematic review on digital mental health in China and found that DHTs were generally feasible and acceptable among people with mental health problems, including people diagnosed with schizophrenia [29].

Among different types of DHTs, smartphones are the most advantageous tool for symptom monitoring given their ability to generate a plethora of data through active and passive monitoring methods [14]. Active monitoring is typically defined as the user self-reporting data (eg, psychological, behavioral, physiological) by ecological momentary assessment or other ambulatory assessment methods [12]. Passive monitoring refers to using the smartphone and its embedded sensors or a wearable device to measure the user’s behavior pattern (eg, app usage, activity log) or contextual information (eg, location) via passive user data collection [36]. By combining active and passive monitoring, smartphone-based monitoring has the potential to provide a more dynamic, personal, and valid representation of a person’s emotional and behavioral state [16,37]. Additionally, studies have shown that active self-monitoring of symptoms can reduce positive psychotic symptoms in people with recent-onset psychosis [38].

Smartphone-based symptom monitoring for people with psychosis has proved to be feasible in the Western context [39]; however, only 1 study conducted in non-Western, low and middle-income countries tested a digital platform with a symptom monitoring element [40]. To date, no smartphone-based symptom monitoring tool has been developed and evaluated for people with psychosis in China [29].

### Study Objectives

The aims of this study are to evaluate the validity, feasibility, acceptability, and safety of a symptom self-monitoring smartphone app (YouXin) for people with psychosis in China.

## Methods

### Ethical Considerations

The study was approved by the University of Manchester (Ref: 2022-13262-24297) and Beijing Anding Hospital (Ref: [2021] Research No.58 ) Research Ethics Committee. The study was conducted in full conformance with all relevant legal requirements and the principles of the Declaration of Helsinki, Good Clinical Practice, and the UK Policy Framework for Health and Social Care Research 2017. Potential participants were given a participant information sheet to read. The detailed informed consent procedure is described in the Recruitment section. Participants were informed that they are free to withdraw from the study at any time and for any reason. Withdrawal from the study did not impact participants' access to standard care at Beijing Anding Hospital. The delivery of the intervention did not affect any form of clinical care participants received, and they continued to be actively supported by the hospital during their participation in the study. Study data are anonymous and were managed according to the data management plan described in the Data Management section. Participants were given a ¥100 (US $13.76) gift voucher for each completed assessment. For participants who took part in the interview, at the end, they were given a ¥100 (US $13.76) gift voucher as a thank you for their time and to cover any costs incurred as a result of their participation. This information was included in the participant information sheet and was provided to participants before they entered the study.

### Study Setting

The study took place at Beijing Anding Hospital of Capital Medical University, which is one of the largest tertiary psychiatric hospitals in the Beijing metropolitan area. The hospital has 800 beds, an average 5000 inpatient admissions per year, and over 1200 daily outpatient visits.

### Study Design

The study uses a mixed methods design consisting of a nonrandomized feasibility study and a nested qualitative acceptability study. The study was designed according to the Consolidated Standards of Reporting Trials (CONSORT) statement extension for pilot and feasibility studies [41]. We recruited 40 people with psychosis at a tertiary-level psychiatric hospital in Beijing, China. This number is in line with other feasibility studies and sufficient to report on response rates, follow-up rates, safety information, and attrition, as well as the clinical characteristics of our study population at the beginning of the study and at follow-up [42]. Formal power calculations for treatment effects are not appropriate for a study primarily aimed at establishing feasibility. This sample size means a monthly recruitment target of 10 participants. Moreover, monitoring the recruitment target is part of the feasibility outcomes.

### Eligibility Criteria

Eligibility criteria were deliberately inclusive to fully assess feasibility and validity of the app and to reach generalizable conclusions for a wide range of service users with psychosis. Due to the limited resources, the research team could not provide devices to participants who did not own a smartphone. However, according to a recent survey study in China, 83.2% of service users and family members reported owning and frequent use of mobile devices [34]. Participant inclusion and exclusion criteria are presented in Textbox 1.

**Inclusion and exclusion criteria.**

**Inclusion criteria:**

- Diagnosed with schizophrenia spectrum according to DSM-5 (F20-F29) or met the criteria for being at clinically high risk for developing psychosis according to the Structured Interview for Prodromal Syndromes (SIPS)
- Aged 16 years to 65 years
- Receiving care from Beijing Anding Hospital and will continue to be actively supported by the hospital over the next 5 weeks (ie, the duration of the project’s participant enrollment period)
- Own and able to use a smartphone

**Exclusion criteria:**

- Diagnosed with organic or substance-induced psychosis
- Lack of capacity to provide informed consent
- Unsafe to participate in research as viewed by their treating clinician (ie, clinically unstable or at risk for self-harm or harm to others)

### Recruitment

Participants were recruited from Beijing Anding Hospital, which is a major tertiary psychiatric hospital located in Beijing, China. Participants were initially approached by a clinician working at the hospital to introduce the project and assess the willingness and potential eligibility of the potential participant to participate in the study. If participants appeared to be eligible and willing to participate, they were referred to the principal researcher who confirmed their eligibility. When eligible, potential participants were given a participant information sheet to read, and their contact details were obtained by the researcher with their consent. The principal researcher followed up with a phone call to the potential participant after at least 48 hours to explain the details of the study and answer any questions that the potential participant had about the study. If the potential participant was still interested in taking part, a face-to-face meeting was arranged to obtain written consent to participate using a hard copy consent form prior to conducting the baseline assessment at the same meeting. Participants were recruited until the recruitment target was met (n=40).

### Study Intervention and Procedures

**Figure 1:**
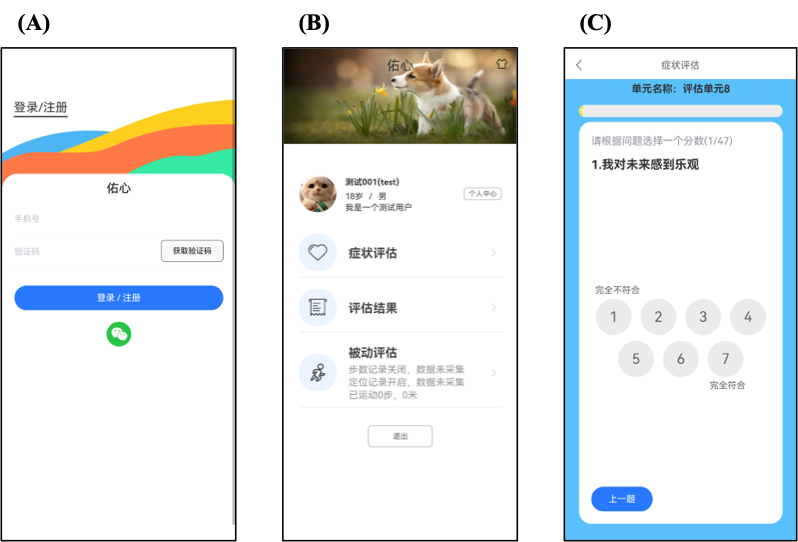
Screenshots of the app prototype: (A) log-in page, (B) user center, (C) assessment module.

YouXin is a smartphone app designed to facilitate symptom self-monitoring for people with psychosis. The app is adapted from the ClinTouch app [13], a self-monitoring app for psychosis validated against the Positive and Negative Syndrome Scale (PANSS) and the Calgary Depression Scale for Schizophrenia (CDSS) items. The PANSS and CDSS have been translated and validated in Chinese and have been widely used in practice in China [43,44]; however, app-based versions of these measures among people with psychosis have not been validated in Chinese. In this study, we translated the ClinTouch-based PANSS and CDSS items into Chinese and combined these items with passively collected data to develop the YouXin app to be tested in the Chinese context. Screenshots of the YouXin app are shown in Figure 1.

The YouXin development team consisted of experts from various disciplines, including academics, clinicians, software engineers, and experts by experience. The principal researcher led the development of the app and was supported by members of the research team who are academic clinicians at the University of Manchester (a Professor of Clinical Psychology and a Professor of Adult Psychiatry). A psychiatrist at Beijing Anding Hospital assisted the development of the app to ensure the content reflected the Chinese context. A technology team led by a computer scientist at Beijing Jiaotong University supported software development.

**Figure 2:**
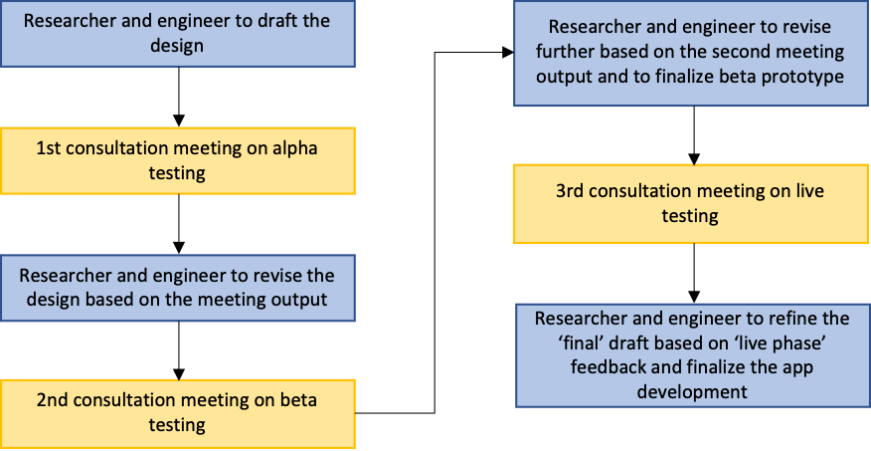
The coproduction process.

To maximize the usability and acceptability of the app for end users (ie, service users and clinicians), the development process utilized a systematic coproduction approach. A series of coproduction consultation meetings was conducted by the principal researcher with service users and clinicians at Beijing Anding Hospital. We used an iterative approach to coproduction, which means the outputs of a previous meeting formed the content of subsequent consultation meetings. The framework for coproduction (shown in Figure 2) was inspired by the process of development used for the Actissist app [45,46], a cognitive behavioral therapy–informed app designed to support people in the early phase of psychosis, and the UK government user research guidelines [47]. A series of participatory design consultations involving 3 phases (alpha, beta, and live phases) was held with service users with severe mental health problems and clinicians. As the YouXin app is adapted from the ClinTouch app, we skipped the discovery phase, which is normally used to generate ideas and hypotheses for a completely new product. The remaining 3 phases are described in Textbox 2.

**The phases of the coproduction process.**

Alpha:

- The alpha phase aims to understand the end users’ (clinicians and service users) needs for using digital tools to monitor symptoms and demonstrate the preliminary design with users (the ClinTouch app and the user interface prototypes of the YouXin app). The design, contents, and functions of the app were further developed based on the feedback from users.

Beta:

- The principal researcher and software engineer integrated the feedback and delivered a functional prototype that users could install on their phones. During the meeting, users tested the app and provided feedback.

Live:

- A fully developed digital platform was presented to users (3 clinicians and 3 people with psychosis) to try out for a week. This meeting focused on evaluating the usability and acceptability of the app and any major issues regarding implementation.

YouXin has 2 core functions: active monitoring of symptoms (ie, smartphone survey) and passive monitoring of behavioral activity (ie, passive data collection via embedded smartphone sensors). The app is available for both Android and iOS platforms. The active monitoring uses a time-contingent design. To minimize participant assessment burden and increase compliance, in accordance with a previous similar study [38], prompts were set to alert at 2 pseudorandomized timepoints per day, during a 12-hour interval from 10 AM to 10 PM, 7 days a week for 4 weeks. A minimum 1-hour increment was set between each prompt on a particular day to avoid clustering of responses. Participants could either snooze (5 minutes or 30 minutes) or decline answering the prompt. Based on previous works by the research group, on average, it takes 70 seconds to complete each prompt. Smartphone sensors passively collected GPS data every 5 minutes. According to a recent systematic review [39], passive sensing was feasible for measuring negative symptoms; specifically, GPS indicators were associated with apathy, avolition, asociality, and anhedonia [48,49]. All collected data were uploaded to a secure server at Beijing Anding Hospital.

Participants were given a training session by the principal researcher to help them navigate the app after they consented to take part. During the training, the principal researcher helped the participant download the app onto their smartphone and gave a demonstration of the app. Participants had the opportunity to practice using the app and ask questions during the process; they had access to the app from the training session until the 4-week timepoint. An opt-out approach was applied to give participants autonomy to switch on or off the passive sensing section during the study.

The principal researcher provided technical support as needed during the study; no clinical care was provided by the research team (clinical care continued as usual from the service). The participant was referred to their treating clinician for appropriate mental health care should any clinical issues be raised to the research team. Participants were informed at the training sessions that the app is not suitable for seeking urgent medical care while in any emergency circumstance.

### Data Collection

**Figure 3:**
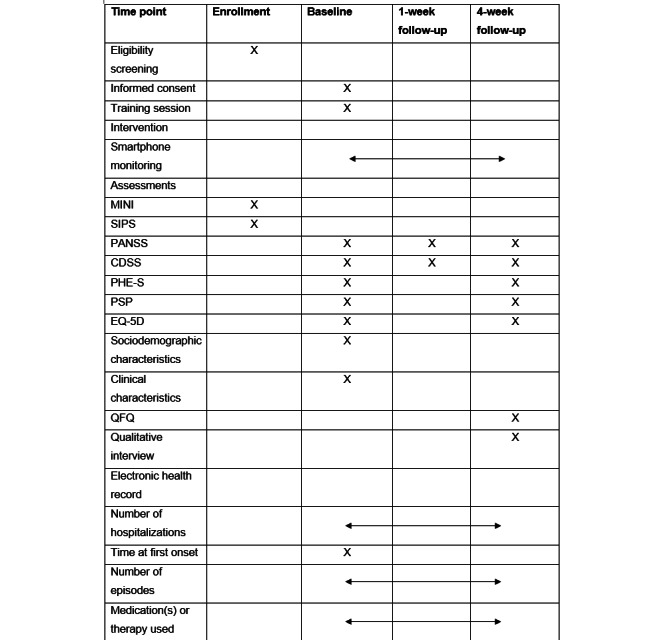
Timeline of data collection. CDSS: Calgary Depression Scale for Schizophrenia; MINI: Mini International Neuropsychiatric Interview; PANSS: Positive and Negative Syndrome Scale; PHE-S, Patient Health Engagement Scale; PSP, Personal and Social Performance; QFQ: quantitative feedback questionnaire; SIPS: Structured Interview for Prodromal Syndromes.

The timeline of outcome assessments is summarized in Figure 3. Participants were assessed in an in-person meeting at baseline, 1 week after baseline, and 4 weeks after baseline. To assess the validity of the remote monitoring, PANSS and CDSS were assessed at 1 week to calculate the association between the active and passive monitoring results and relevant domains of the clinical assessment. To determine which outcomes are sensitive to change in this population, participants were asked to complete an assessment of relevant outcomes of the self-monitoring intervention at baseline and the 4-week timepoint. The assessor is a clinician, has been trained in the assessment measures used, and has rich experience in conducting assessments in clinical trials. Semistructured qualitative interviews were conducted after the 4-week timepoint to explore the acceptability of the app.

### Outcome Measures

#### Sociodemographic and Clinical Characteristics

Sociodemographic characteristics including age, gender, education level, marriage status, average income, accommodation and living situation, employment status, and ethnicity were collected. We also collected data regarding digital literacy and mental health literacy.

#### Data From Service Users’ Electronic Health Records

Number of hospitalizations, time at first onset, number of episodes, and medication(s) or therapy used before and during the study were collected from service users’ electronic health records (EHRs). At Anding Hospital, the EHR is used in both outpatient clinics and inpatient units and is shared within the hospital. All records are kept in the EHR system using computers, and no records are written by hand; service users receive a hard copy of their record following each appointment.

#### Clinical Measurements

The Chinese version of the Mini International Neuropsychiatric Interview [50,51] was used to determine the DSM-5 diagnosis.

The Chinese version of the Structured Interview for Prodromal Syndromes [52,53] was used to assess the risk for psychosis.

Psychotic symptoms were rated using the Chinese version of the PANSS [54,55], a 30-item scale that assesses positive symptoms, negative symptoms, and general psychopathology. The Chinse version is comparable to the original version and has good internal consistency reliability (Cronbach α=.87) [43].

Mood symptoms were assessed using the Chinese version of the CDSS [56,57], a 9-item scale designed specifically for assessing depression for schizophrenia. The Chinese version [44] has good reliability (Cronbach α=.798-.810) and is significantly correlated with the Hamilton Depression Scale (r=0.76) and PANSS-G6 (r=0.70).

Service user engagement was measured using the Chinese Version of the Patient Health Engagement Scale [58], a self-report scale assessing service users’ attitudes toward engagement in health care.

Social functioning was rated using the Chinese version of the Personal and Social Performance scale (PSP) [59,60], a 100-point single-item rating scale assessing 4 main areas of functioning, including socially useful activities, personal and social relationships, self-care, and disturbing and aggressive behaviors.

Quality of life was evaluated using the Chinese version of the EQ-5D-5L [61], a self-report questionnaire designed to assess health status and health-related quality of life. The EQ-5D-5L consists of 5 questions that assess 5 health dimensions and a visual analog scale to assess the overall current health judged by the individual respondents.

#### Smartphone Monitoring

##### Active Monitoring

Active monitoring consisted of the following 3 sections: psychotic symptoms, mood symptoms, and contextual information. The psychotic and mood symptom items were translated into Chinese from the ClinTouch app and were compared against the Chinese version of the PANSS and CDSS. Contextual questions were added to understand the context of participant responses and to validate GPS data. Examples of the items are shown in Table 1.

**Table 1:** Examples of the active monitoring items.

Domain	Item	Response items	Format
Psychotic symptoms	“I have heard voices.”	1-7	Likert scale
Mood symptoms	“I have felt sad.”	1-7	Likert scale
Contextual questions	“Where were you?”	At home; at work; at school or other educational setting; in hospital; in vehicle; in a convenience store, supermarket, or shopping mall; outside walking; inside other; outside other	Check box

##### Passive Monitoring

Passive monitoring used collected GPS data to measure mobility and activity level of users. GPS mobility indicators measured on the smartphone, including home time, distance travelled, and location clusters, will be extracted following the procedures by Müller et al [62]. Briefly, locations will be clustered as “unique” if a person spent at least 30 minutes within a 200-meter radius. Home location will be determined by selecting the mode of location clusters during the night (ie, 9 PM to 6 AM). Distance travelled will be computed by aggregating the distance changes between 2 successive GPS data points; however, distance changes of GPS data points in the same location cluster will be considered as zero. Time spent at each location, including time at home, will be calculated by summing the differences of the time stamps of 2 successive GPS data points in the location cluster.

#### Validity

The validity of active monitoring items will be assessed against relevant clinical assessments (ie, PANSS and CDSS) using Spearman correlation. An exploratory analysis will also be conducted to understand the association between mobility indicators, corresponding contextual questions in active monitoring, and PANSS negative symptom scores. We define a moderate statistically significant correlation (ie, Spearman rho>0.35) as the validity threshold.

#### Feasibility Parameters

To assess the feasibility of the study, we collected detailed recruitment and retention data accordant with the CONSORT statement for feasibility studies [41], including (1) number of eligible participants consenting; (2) completeness of outcome measures; (3) number of participants lost to follow-up; and (4) reason for withdrawal. We defined Green (>80%; ie, feasible and no changes needed), Amber (60%-80%; ie, additional elements required to increase feasibility), and Red (<60%; ie, not feasible) categories to indicate the levels of feasibility for the above parameters. We will explore app usage and engagement data guided by the Analyzing and Measuring Usage and Engagement Data framework [63].

#### Acceptability

The quantitative feedback questionnaire (QFQ) was given to participants at the 4-week timepoint to collect quantitative information regarding acceptability. The QFQ is a 27-item questionnaire designed to assess the acceptability of utilizing smartphone app interventions for service users. It has been used in previous digital mental health studies for people with psychosis [64,65].

Qualitative interviews were conducted to explore their experiences using Youxin including (1) overall impressions of YouXin, (2) positive and negative aspects of the app in terms of content and usability, (3) how it helped or did not help, (4) what changes they would make, (5) barriers and facilitators to engagement, and (6) views on the training session.

#### Safety

To evaluate the safety of the app, detailed adverse events and serious adverse events were collected and reported using standardized operating procedures in line with National Institute for Health and Care Research and Health Research Authority Safety Reporting procedures.

### Safety Considerations, Adverse Events, and Risks

We did not expect participating in the study would cause any significant risks. Although the study involves people with severe mental health problems, the eligibility criteria for the study require the participants to be stable and at low risk for self-harm or harm to others, as judged by a clinician. There is the potential for participants to report elevated levels of distress because of taking part in assessments and digital self-monitoring. This may include the potential for increasing awareness of one's own symptoms. Participants were informed during the recruitment process that they may encounter such situations, and they were encouraged to discuss this with the research team if they feel distressed during the study.

### Statistical Analysis Plan

All quantitative analyses will be performed using R (version 4.0.5) [66]. Feasibility data and safety data including (1) recruitment and retention data, (2) app usage data, (3) completeness of study measures, and (4) the number and nature of adverse events will be analyzed using appropriate descriptive statistics (mean and standard deviations for continuous data; counts and percentages for categorical data). Logistic regression and linear regression analyses will be used to explore sociodemographic and clinical characteristics and device attributes as predictors of feasibility (eg, dropouts, app usage).

The active monitoring will generate 3 levels of data, with the data entered for each “beep” as level 1, nested within the level 2 “day” level, which is nested within the level 3 subject level. Because the PANSS and CDSS measure participants’ symptoms for the past week, and to minimize the impact of missing data, the subject level mean score of the first week will be used to calculate the association between active monitoring items score and relevant symptom domain scores of the PANSS and CDSS at the week 1 follow-up assessment to evaluate validity. The significance level for the study will be set to .05. Cronbach alpha will be used to calculate the internal consistency reliability of the sets of the items of active monitoring. To account for the nested structure of the data, multilevel models will be used to explore the instability of the different constructs across time and patterns of missing data.

The GPS mobility indicators will be extracted from passive monitoring data. The convergent validity of passive monitoring will be calculated by the correlation between GPS mobility indicators and the PANSS negative symptom score. Discriminant validity of passive monitoring will be demonstrated by low correlation between GPS mobility indicators and positive symptoms measured by PANSS.

Qualitative data will be transcribed verbatim in Chinese and then translated to English by the principal researcher. A framework analysis approach will be used based on the protocol by Braun and Clarke [67], which consists of the following 6 phases of thematic analysis: familiarization, transcription, generating initial codes, searching for themes, reviewing themes, and defining and deciding on meaningful themes. Nvivo software (version 12) [68] will be used to manage the qualitative data.

### Data Management

Names and contact information are password-protected and stored electronically and separately with anonymous questionnaire responses in a secure, password-protected folder on the principal researcher’s university drive and are kept for the purposes of contacting participants during the study. All quantitative data are stored as an anonymous electronic file and stored on the principal researcher's university drive for the purpose of analysis. Data generated from the smartphone app are stored on a secure server at Beijing Anding Hospital. The server is managed by the IT department of Beijing Anding Hospital according to the Chinese government’s data protection regulations. Only the IT staff can access the data stored on the server. The principal researcher applied to download the raw data at the end of the study data collection.

Qualitative interview data recordings were anonymized as soon as practical after the interview, and anonymous transcripts were prepared by the principal researcher. Personal information was removed from the final transcripts or replaced with pseudonyms where appropriate. The file name was saved with the date of transcription and allocated a number to protect confidentiality and ensure no file names are the same (ie, DD_MMM_YYYY_SU_1). For the publication write-up, quotations may be used for which the participants will be given a pseudonym (ie, “service user1,” which will be representative of the allocated number of the saved document name); however, they will not be identifiable by the research team at this point.

## Results

Recruitment for the study was initiated in August 2022. Of the 47 participants who were approached for the study from August 2022 to October 2022, 41 participants agreed to take part in the study. Reasons for not participating included concerns that answering app questions would be a burden (n=1) and family member concerns that answering app questions might be triggering for the participant (n=1). Another 4 participants declined to participate without giving any reason. We excluded 1 participant for not meeting the inclusion criteria, leaving a total of 40 participants taking part in the study. As of December 2022, 40 participants had completed the study. We expect to report the validity of the app-based monitoring and results for feasibility on the (1) number of eligible participants consenting, (2) completeness of outcome measures, (3) number of participants lost to follow-up, and (4) reasons for withdrawal. We also expect to report participants’ overall impressions of YouXin, positive and negative aspects of the app in terms of content and usability, perceived helpfulness of the app, barriers and facilitators to engagement, and views on the training session.

## Discussion

This study aims to test the validity, feasibility, acceptability, usability, and safety of the YouXin smartphone app for people with psychosis in China to self-monitor symptoms. To the best of our knowledge, this study is the first to develop and test a symptom self-monitoring app specifically designed for people with psychosis in China. Therefore, a feasibility study is necessary to determine if a DHT like this will be appropriate to facilitate self-management for this population.

We conducted a systematic review and series of surveys and qualitative interviews with people with severe mental health problems and mental health staff in China to facilitate the development of the app. Based on our previous findings, DHTs were generally feasible and acceptable among people with mental health problems in China [29], and mental health professionals were willing to implement such technologies in clinical practice [30]. In addition, DHTs developed in China have rarely used the coproduction approach [29]; we coproduced the app to maximize the chance it meets end user needs.

One of the concerns of utilizing DHTs is the risks related to data security and privacy, which may hinder the implementation of DHTs [69-71]. To tackle these issues, we designed and developed the app to meet the standard required by China’s Data Security Law. Additionally, we discussed these issues with both the service users and mental health staff in the coproduction process, and the feedback from the coproduction process indicated that, as long as the app was designed and developed according to data security regulations, both staff and service users felt safe using it. Moreover, we conducted postintervention interviews with participants to have an in-depth understanding of their concerns and how data security and privacy issues may impact their usage of the app.

If YouXin is feasible, acceptable, and safe and brings clinical benefit, a potential future direction is to integrate the app into clinicians’ workflows to facilitate a digitally enabled care pathway to support timely mental health care for people with psychosis in China. This could include a remote symptom monitoring system [38] or a more comprehensive integrated digital clinic [35]. According to views collected from mental health staff during the coproduction workshops, staff expressed positive attitudes toward implementing digital mental health care in practice and considered digital health to be the future of mental health care.

In summary, this study will provide valuable insights for evaluating the feasibility, safety, acceptability, trial procedures, and implementation strategies of a DHI with this population in the Chinese context. Moreover, the findings of this trial have the potential to inform the development of digital solutions for China’s mental health services in general, and, more specifically, it could lead to optimization of DHTs designed for people with psychosis in China.

## Abbreviations

CDSS: Calgary Depression Scale for Schizophrenia
CONSORT: consolidated standards of reporting trials
DHT: digital health technology
DSM: diagnostic and statistical manual of mental disorders
EHR: electronic health record
PANSS: Positive and Negative Syndrome Scale
QFQ: quantitative feedback questionnaire

## Data Availability

The data sets generated during or analyzed during this study are available from the corresponding author on reasonable request.
